# Socioeconomic aspects of incretin-based therapy

**DOI:** 10.1007/s00125-023-05962-z

**Published:** 2023-07-12

**Authors:** Thomas Karagiannis, Eleni Bekiari, Apostolos Tsapas

**Affiliations:** 1 Clinical Research and Evidence-Based Medicine Unit, Second Medical Department, Aristotle University of Thessaloniki, Thessaloniki, Greece; 2 Diabetes Centre, Second Medical Department, Aristotle University of Thessaloniki, Thessaloniki, Greece; 3 Harris Manchester College, University of Oxford, Oxford, UK

**Keywords:** DPP-4 inhibitors, GLP-1 receptor agonists, Healthcare equity, Incretin, Incretin-based therapy, Review, Socioeconomic status, Type 2 diabetes

## Abstract

Incretin-based therapies, particularly glucagon-like peptide-1 receptor agonists (GLP-1 RAs), have demonstrated cardiovascular benefits in people with type 2 diabetes. However, socioeconomic disparities in their uptake may constrain the collective advantages offered by these medications to the broader population. In this review we examine the socioeconomic disparities in the utilisation of incretin-based therapies and discuss strategies to address these inequalities. Based on real-world evidence, the uptake of GLP-1 RAs is reduced in people who live in socioeconomically disadvantaged areas, have low income and education level, or belong to racial/ethnic minorities, even though these individuals have a greater burden of type 2 diabetes and cardiovascular disease. Contributing factors include suboptimal health insurance coverage, limited accessibility to incretin-based therapies, financial constraints, low health literacy and physician–patient barriers such as provider bias. Advocating for a reduction in the price of GLP-1 RAs is a pivotal initial step to enhance their affordability among lower socioeconomic groups and improve their value-for-money from a societal perspective. By implementing cost-effective strategies, healthcare systems can amplify the societal benefits of incretin-based therapies, alongside measures that include maximising treatment benefits in specific subpopulations while minimising harms in vulnerable individuals, increasing accessibility, enhancing health literacy and overcoming physician–patient barriers. A collaborative approach between governments, pharmaceutical companies, healthcare providers and people with diabetes is necessary for the effective implementation of these strategies to enhance the overall societal benefits of incretin-based therapies.

**Graphical Abstract:**

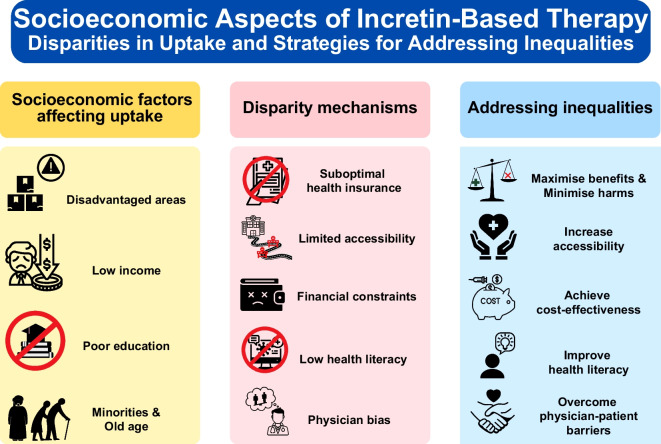


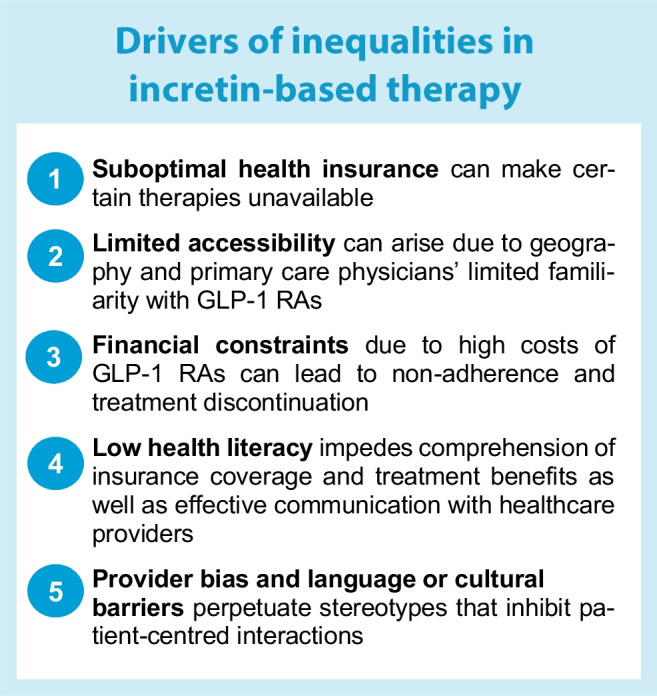



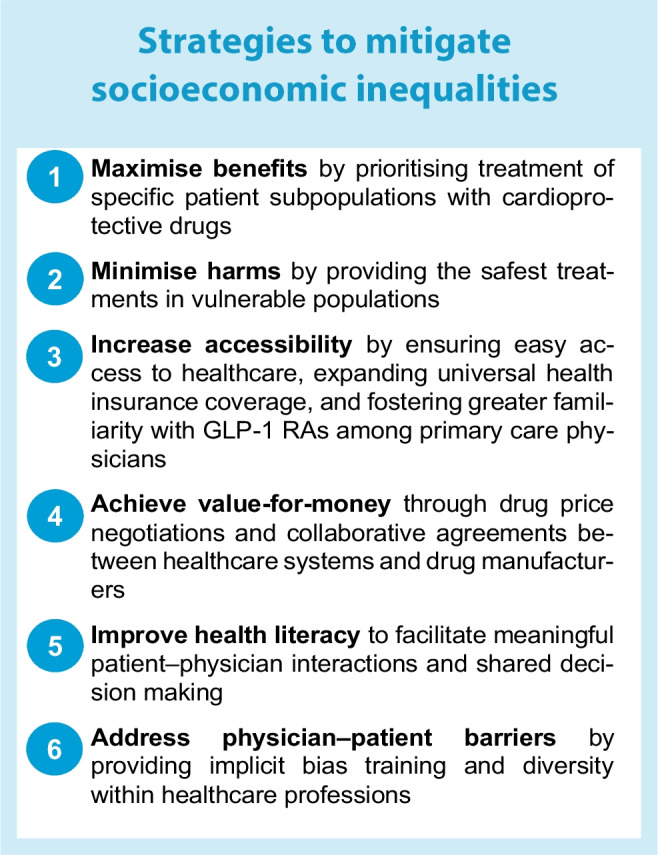


## Introduction

Socioeconomic status refers to the social and economic factors that influence what position individuals or groups hold within the structure of a society [1]. These factors commonly include housing, income, education level and occupation, while non-modifiable sociodemographic characteristics, such as race/ethnicity or age, are also important variables to be considered in the relationship between socioeconomic status and health [2, 3]. Low socioeconomic status is considered a strong and consistent predictor of a person’s morbidity and mortality and is an important risk factor for type 2 diabetes both in high-income and low-income countries [4, 5]. Mediators linking low socioeconomic status with type 2 diabetes include obesity, alcohol consumption, reduced physical activity, psychosocial stress, low health literacy or limited access to healthy food and exercise facilities [5, 6]. Low socioeconomic status in individuals with type 2 diabetes can result in poor management of metabolic variables [3, 7] and is associated with increased mortality and risk for cardiovascular complications [2, 8–13].

Disparities in diabetes care can be affected by socioeconomic status, which may also lead to unequal utilisation of cardioprotective treatments [3]. Incretin-based therapies are a group of anti-hyperglycaemic drugs including glucagon-like peptide-1 receptor agonists (GLP-1 RAs), dipeptidyl peptidase 4 (DPP-4) inhibitors, and recently developed dual glucose-dependent insulinotropic polypeptide (GIP)/GLP-1 RAs such as tirzepatide [14]. The ADA and EASD recommend use of agents that have demonstrated cardiovascular benefits in diabetic individuals with elevated cardiovascular risk [14]. Certain GLP-1 RAs are included among these agents [14], and emerging data suggest that tirzepatide may also have cardioprotective effects [15].

In this review, we summarise real-world evidence regarding the use of incretin-based therapies in clinical practice across the socioeconomic spectrum, while exploring the possible drivers behind socioeconomic disparities in the adoption of these therapies. Moreover, we examine approaches to enhance societal benefits through their optimal use by factoring in aspects beyond their effect on cardiovascular outcomes, such as their safety profile and cost-effectiveness from a broad societal perspective.

## Utilisation of incretin-based therapy based on socioeconomic status

To facilitate identification of studies assessing the effect of socioeconomic status on utilisation of incretin-based therapies, we searched PubMed in January 2023 adapting a previously used search strategy [16], using the following search terms: (("socioeconomic factors" or "social class" or "socioeconomic" or "social" or "income" or "education*" or "depriv*") AND ("glucagon-like peptide 1" or "glp1*" or "glp-1*" or "glucagon-like-peptide-1" OR "dpp4" or "dpp-4" or "dipeptidyl peptidase 4" or "dipeptidyl peptidase-4" or “incretin”)) AND (type 2 diabetes). Most pertinent studies, mainly retrospective cohort studies, were conducted in the USA, whereas assessed socioeconomic factors varied across studies, including area-level indexes, income, education or sociodemographic variables (Table 1).

**Table 1:** Summary of key findings of cohort studies assessing the effect of socioeconomic factors on utilisation of incretin-based therapies

Country	Study	Number of participants	Factors	Key findings
Australia	Morton 2021 [18], Morton 2022^a^ [19]	1,203,317	• Index of Relative Socioeconomic Disadvantage	• People in disadvantaged areas less likely to receive GLP-1 RAs (OR vs more advantaged areas ranging between 0.72 and 0.95)
Denmark	Falkentoft 2022 [16]	48,915	• Income • Education • Race/ethnicity	• High-income patients more likely to initiate GLP-1 RAs (PR vs low-income patients, 1.24) • Higher education linked to higher GLP-1 RA initiation (PR vs lower education levels ranging between 1.08 and 1.19) • Observed disparities more pronounced in immigrant/descendant subgroup
UK	Whyte 2019 [20]	84,452	• Index of Multiple Deprivation • Race	• Most socioeconomically deprived group less likely to receive GLP-1 RAs (OR vs least deprived group, 0.89) and more likely to receive DPP-4 inhibitors (OR, 1.13) • Asian (OR vs White patients, 0.55) and Black (OR, 0.45) patients less likely to be prescribed GLP-1 RA. Asian (OR, 1.29) patients more likely to be prescribed DPP-4 inhibitor
USA	Cromer 2023 [17]	4,057,725	• A USA social deprivation index [27] • Age	• Higher deprivation linked to lower GLP-1 RA usage among patients with atherosclerotic disease (HR vs lower deprivation, 0.94) • Older age linked to lower GLP-1 RA usage (HR per year of age, 0.94)
	Eberly 2021 [21]	1,180,260	• Income • Race/ethnicity	• Higher income linked to higher GLP-1 RA usage (OR vs lower incomes ranging between 1.07 and 1.13) • Asian (OR vs White patients, 0.59), Black (OR, 0.81) and Hispanic (OR, 0.91) patients less likely to receive GLP-1 RA
	McCoy 2021 [22]	382,574	• Income • Race/ethnicity • Age	• Lower income linked to decreased GLP-1 RA initiation (OR vs higher income levels ranging between 0.81 and 0.90) • Racial disparities in GLP-1 RA usage in Asian, Black and Hispanic minorities—most pronounced in Asian patients (OR vs White patients, 0.49) • Older age linked to lower GLP-1 RA (OR per year of age, 0.92) and DPP-4 inhibitor usage (OR, 0.95)
	Deniveni 2022 [23]	81,332	• Income • Education	• Higher income patients more likely to be on GLP-1 RA treatment (OR vs low income, 1.28) • College-educated patients more likely to be on GLP-1 RA treatment
	Vasti 2023 [25]	793,525	• Education	• Low education linked to lower GLP-1 RA usage (OR vs higher education, 0.85)
Global (37 countries across six regions)	Nicolucci 2019 [26]	14,668	• Education	• Lower education linked to lower usage of GLP-1 RA (OR vs higher education levels ranging between 0.32 and 0.33) or DPP-4 inhibitor (OR ranging between 0.48 and 0.70)

PR, probability ratio

^a^ Morton 2022 is a follow-up of Morton 2021

### Area-level indexes

A study in the USA found that, among individuals with type 2 diabetes and cardiovascular disease, those with increased area-level socioeconomic deprivation were less likely to receive GLP-1 RAs compared with those living in more privileged areas [17]. A similar trend was observed for sodium–glucose cotransporter 2 (SGLT-2) inhibitors, although the disparity was not as pronounced as with GLP-1 RAs [17]. In Australia, a study assessing the relationship between treatment with newer glucose-lowering medications and the Index of Relative Socioeconomic Disadvantage, found that individuals living in the most socioeconomically disadvantaged areas were consistently less likely to receive GLP-1 RAs, whereas the opposite was observed for DPP-4 inhibitors [18, 19]. Of note, the Index of Relative Socioeconomic Disadvantage ranks areas in Australia based on information about income, education, employment, occupation, housing and other indicators [18]. This study also found a connection between low socioeconomic status and a reduced probability of receiving SGLT-2 inhibitors, albeit less marked than that observed with GLP-1 RAs, while no such relationship was identified for metformin, sulfonylureas or insulin [18, 19]. According to a UK study, when fully accounting for various confounding factors, individuals belonging to the most socioeconomically deprived group, as identified by the Index of Multiple Deprivation (an overall relative measure of deprivation in England which is based on seven socioeconomic domains), had a lower likelihood of being prescribed GLP-1 RAs [20]. Conversely, these individuals were more likely to receive DPP-4 inhibitors in comparison with their counterparts in the least deprived group [20].

### Income

In a USA study, the odds of receiving treatment with a GLP-1 RA were higher in individuals with diabetes who had a high household income compared to those with an annual income of less than US$50,000, and these findings were consistent in a subgroup analysis comprising solely participants with established cardiovascular disease [21]. In another USA study, low annual household income was also associated with decreased odds of initiating a GLP-1 RA, whereas this association was not observed for DPP-4 inhibitors, likely owing to their lower out-of-pocket expenses compared with GLP-1 RAs [22]. Higher income individuals were also more likely to be on GLP-1 RA treatment in a USA contemporary cohort study (*All of Us* Research Program) [23]. In Denmark, metformin-treated patients with a high household income were more likely to initiate second-line treatment with a GLP-1 RA compared to those with low household income, and this finding was consistent in a subgroup analysis irrespective of presence of cardiovascular disease [16]. Notably, studies have also reported an association between low income and decreased odds of SGLT-2 inhibitor treatment, although this relationship was less pronounced than the findings for GLP-1 RAs [16, 22, 24].

### Education

Education is a frequently used indicator of socioeconomic status, which captures the knowledge-related assets of a person and is a determinant of future employment, occupation and income [1]. In the USA, diabetic individuals with a high school level education had lower odds of receiving a prescription of GLP-1 RA or SGLT-2 inhibitor in comparison to those with a postgraduate degree [25]. Similarly, in the *All of Us* Research Program, a higher percentage of diabetic individuals who went to college were on GLP-1 RA treatment compared to those with less than a high school diploma [23]. In Denmark, the probability of initiating either a GLP-1 RA or an SGLT-2 inhibitor as second-line therapy was higher in those with college education compared with lower educational levels [16]. Furthermore, multinational data from the global DISCOVER programme suggested that people with an education duration of less than 13 years had lower odds of receiving a GLP-1 RA rather than a sulfonylurea [26]. Similar, albeit less marked, associations with education level and drug utilisation were also found for DPP-4 inhibitors and SGLT-2 inhibitors, but not for insulin [26].

### Sociodemographic factors

Sociodemographic factors such as race/ethnicity and age can also influence the uptake of newer medications and have been taken into consideration in the development of socioeconomic indexes [27]. Retrospective cohort data from the USA suggest that, compared to White individuals with type 2 diabetes, Asian, Black and Hispanic individuals were less likely to receive GLP-1 RA therapy [21]. Notably, this was also the case for SGLT-2 inhibitors in a similar cohort [24]. Consistent findings were observed in another USA cohort, where racial inequalities regarding GLP-1 RA use were evident in Asian, Black and Hispanic minorities [22]. Asian participants were also less likely to receive SGLT-2 inhibitor therapy compared with White participants, while use of DPP-4 inhibitors did not differ across races/ethnicities [22]. In the UK, compared with White individuals, Asian and Black minorities were more likely to be prescribed metformin or sulfonylureas instead of GLP-1 RAs or SGLT-2 inhibitors, while Asian participants were more likely to receive DPP-4 inhibitors [20]. In Denmark, inequalities in GLP-1 RA therapy between diabetic individuals with a high income and those with a low income were more pronounced in the immigrant/descendant subgroup than in the native Danish population [16]. Older age in the USA has been associated with lower probability of receiving GLP-1 RA or SGLT-2 inhibitor in people with type 2 diabetes and atherosclerotic cardiovascular disease [17]. Similar findings were observed in another USA study, both for GLP-1 RAs and for DPP-4 inhibitors [22].

## Mechanisms of socioeconomic inequalities in utilisation of incretin-based therapy

The text box ‘Drivers of inequalities in incretin-based therapy’ summarises some key mechanisms that often interact with each other to drive socioeconomic inequalities in the utilisation of incretin-based therapies.

### Suboptimal health insurance coverage

Policies on health insurance coverage can vary considerably between countries; countries with universal healthcare systems may exhibit different patterns of access and affordability in comparison to countries with predominantly private healthcare systems [28]. In a system providing universal reimbursement, as exemplified by many European countries, access to incretin-based therapies may be more equitable as the treatments are often available to a larger proportion of the population, irrespective of an individual’s financial status. However, despite overall well-regulated medication prescription practices in Europe, access to GLP-1 RAs is not consistent across European populations owing to between-country policy variations. For example, GLP-1 RAs in the UK can only be prescribed to people with type 2 diabetes who also have obesity, while many other European countries do not impose such restrictions [28].

In a system without universal insurance coverage, individuals from lower socioeconomic backgrounds may face more substantial barriers to accessing these therapies due to higher out-of-pocket costs or limited insurance coverage [28]. In the USA, although Medicare aims to support low-income individuals and families, not all states have expanded Medicare coverage, leaving many people without access to health insurance. As such, lack of insurance for many people from lower socioeconomic backgrounds in the USA makes it impossible for them to gain access to expensive medications [23]. Notably, socioeconomic disparities can persist even with Medicare coverage, as demonstrated by a study showing that Medicare beneficiaries had significantly lower odds of receiving GLP-1 RAs compared to individuals with private (commercial) insurance [22]. A similar trend was observed in Germany, with private health insurance being a strong predictor of GLP-1 RA prescription [29].

### Limited accessibility

People living in rural or socioeconomically disadvantaged areas may face difficulties in finding and attending medical appointments with professionals who specialise in diabetes care, and may even encounter challenges in accessing pharmacies that offer the latest GLP-1 RAs [18, 26, 28]. In the absence of diabetes specialists, primary care physicians who may not be well-versed in the cardiovascular benefits of GLP-1 RAs could be hesitant to modify therapies when glycaemic control is stable, and feel reluctant to prescribe these medications even for patients with cardiovascular disease [23, 25]. The limited familiarity of primary care physicians with incretin-based therapies can significantly impact accessibility to GLP-1 RAs, given that most people with type 2 diabetes are treated by primary care physicians rather than diabetologists or endocrinologists. In fact, research suggests that diabetologists and endocrinologists are more likely to prescribe GLP-1 RAs than primary care physicians, possibly due to their increased familiarity with injectable incretin-based therapies [18, 23, 26, 29]. Interestingly, a similar association of socioeconomic disadvantage and specialist prescribing was not observed for SGLT-2 inhibitors [16, 19]. The reasons for this difference are unclear, but it could be partly attributed to administration barriers (subcutaneous injection) of GLP-1 RAs [16].

### Financial constraints

Even with health insurance coverage and access to incretin-based therapies, individuals with lower incomes may still find it challenging to afford the high cost of GLP-1 RAs [18, 23, 30–32]. For economically disadvantaged populations, the expense of GLP-1 RAs could be prohibitive, as co-payments for these medications can quickly accumulate, leading many people to opt for less expensive alternatives. Research indicates that high-income individuals are more likely to receive novel drugs earlier due to their ability to afford high out-of-pocket treatments [32–34]. However, it is important to note that factors related to financial barriers, such as manufacturer prices and co-payment levels for incretin-based medications, can vary considerably between countries [28]. High medication costs can also contribute to non-adherence to prescribed therapy. In a survey study involving more than 5000 participants with diabetes, one in seven reported using fewer medications than prescribed due to cost [35]. Individuals who cannot afford to continue their treatment with GLP-1 RAs may skip doses, take smaller doses or discontinue medications entirely, resulting in suboptimal diabetes management. In fact, as opposed to other glucose-lowering medications, high cost has been shown to be a major factor in suboptimal adherence to GLP-1 RA therapy and even in treatment discontinuation [36].

The disparity in use of GLP-1 RAs between high-income and low-income people with type 2 diabetes is further exacerbated by the ongoing global shortage of semaglutide and dulaglutide, partially driven by increased off-label use of these drugs for weight loss [37, 38]. A considerable portion of the drugs’ limited supply has been redirected towards economically privileged individuals seeking weight reduction, regardless of diabetes status. This shift has disproportionately affected lower socioeconomic groups, particularly those with type 2 diabetes and increased cardiovascular burden, who depend solely on affordable reimbursement processes to access these medications.

### Low health literacy

Health literacy is an important mediator between an individual’s socioeconomic status and adoption of health-related behaviours and interventions [37, 38]. Socioeconomically disadvantaged groups have been consistently reported to have lower health literacy compared with more privileged groups, and this disparity in health literacy has been associated with negative health outcomes and decreased uptake of therapeutic and preventive interventions [39]. Among the factors that can contribute to the underutilisation of GLP-1 RAs in individuals with low health literacy are difficulties in navigating and understanding health information, as well as communication barriers with healthcare professionals. These people may have trouble comprehending insurance coverage options, which can result in confusion about the availability of GLP-1 RAs or the actual out-of-pocket costs they may incur [25]. This, in turn, may lead them to avoid or postpone treatment due to concerns about affordability. Additionally, individuals with lower health literacy are more likely to possess limited prior knowledge about the potential cardiovascular benefits of GLP-1 RAs and may encounter difficulties or feel less keen to seek access to, or request referrals for, diabetes specialists. Moreover, during consultations with healthcare providers, these individuals might struggle to voice their concerns or ask relevant questions about GLP-1 RAs, hindering their ability to fully discuss and address concerns regarding specific barriers related to GLP-1 RAs, such as high cost and need for subcutaneous administration [30, 40].

### Provider bias and physician–patient barriers

Provider bias or cultural and language barriers between physician and patient can contribute to socioeconomic inequalities in the uptake of incretin-based therapies. Healthcare providers may exhibit conscious or unconscious biases in their prescribing practices, leading to disparities in GLP-1 RA utilisation. Specifically, they may be less inclined to prescribe these medications to patients from lower socioeconomic backgrounds or minority groups, perceiving them as less compliant to treatment and medical advice, or incapable of affording the out-of-pocket costs of GLP-1 RAs [25]. However, these perceptions held by some physicians may not be accurate and can lead to disparities in care. This bias may stem from stereotypes or preconceived notions about certain populations, which can exacerbate healthcare disparities. Structural racism can further compound this issue, as it often gives rise to systemic barriers and implicit biases that disadvantage marginalised populations [41]. In this context, minority populations may also encounter challenges in accessing healthcare professionals who understand their language and cultural background and are willing to engage in meaningful patient-centred interactions [16, 21, 32].

## Increasing societal benefit from incretin-based therapy

A summary of strategies addressing socioeconomic disparities in uptake of incretin-based therapy is presented in the text box ‘Strategies to mitigate socioeconomic inequalities’.

### Maximising treatment benefits

Maximising the absolute benefits of a treatment involves emphasising its use in subpopulations that are expected to gain the most from its favourable effects [30]. In addition to their impressive glucose-lowering and weight-reduction potential [42, 43], cardiovascular outcomes trials (CVOTs) have shown that GLP-1 RAs reduce cardiovascular outcomes (especially stroke) in people with elevated cardiovascular risk [42, 44, 45]. Subgroup meta-analyses suggest that these benefits occur in clinically relevant subpopulations including racial minorities [46, 47] and older individuals [48], even though representation of participants older than 75 years and of racial/ethnic minorities has been low in type 2 diabetes CVOTs [48–50]. Real-world evidence from cohort studies has also associated GLP-1 RAs with favourable cardiovascular effects compared with other glucose-lowering drugs [51–54]. Hence, there are consistent data supporting use of incretin-based therapies, particularly GLP-1 RAs, in socioeconomically disadvantaged people with type 2 diabetes, considering that such individuals are at increased risk for developing cardiovascular complications [2].

### Minimising treatment harms

Minimising treatment harms in potentially vulnerable populations is just as important as maximising benefits [30]. GLP-1 RAs have been associated with increased risk for gastrointestinal events, which can lead many patients to treatment discontinuation [55, 56]. The consequences of these adverse events can be more pronounced and potentially dangerous in vulnerable populations, especially older individuals. Moreover, treatments that further decrease weight may not be appropriate for older, frail individuals, while, because of their subcutaneous administration, most GLP-1 RAs may not be a practical treatment option for people with visual or cognitive impairments [57]. On the other hand, DPP-4 inhibitors are generally tolerable and safe for use in older people with type 2 diabetes [58, 59] and, as such, they can be an important treatment option in the older/frail population where quality of life is a priority [57, 60]. It should be noted, however, that caution is warranted if saxagliptin or alogliptin are used in individuals with a history of heart failure [61].

### Increasing accessibility

Addressing healthcare access barriers is essential for enhancing the uptake and use of GLP-1 RAs among individuals from lower socioeconomic backgrounds. This may involve implementing practical measures to facilitate access to healthcare services for people living in rural or low-income regions, such as providing transportation services to healthcare appointments, and increasing the availability and retention of both primary care providers and diabetes specialists in underserved areas [19]. Furthermore, enhancing primary care physicians' familiarity with GLP-1 RAs through targeted education, training and resources is important, as most people with type 2 diabetes are treated by primary care physicians rather than diabetologists or endocrinologists. Another important strategy aiming to achieve equitable access to incretin-based therapy would be to expand health insurance coverage for socioeconomically disadvantaged populations to include early access to high-cost cardioprotective GLP-1 RAs at an affordable cost with low out-of-pocket expenses [30, 62].

### Achieving value-for-money

Availability of beneficial drugs at low cost is also important in terms of increasing their value-for-money from a broader societal perspective. This is especially pertinent when considering newer, high-cost incretin-based therapies, such as semaglutide or tirzepatide. A recent cost-effectiveness study in the USA concluded that, as a first-line therapy, the cost of GLP-1 RAs would need to fall by at least 70% to be cost-effective compared with metformin [63]. Moreover, in Australia, the use of GLP-1 RAs at current prices was unlikely to be cost-effective either for primary or secondary cardiovascular prevention, whereas SGLT-2 inhibitors were found to be cost-effective on both occasions [64]. Similarly, a price target analysis for 67 low-income or middle-income countries found that GLP-1 RAs, as opposed to SGLT-2 inhibitors, were overall not cost-effective in these countries [65]. Findings were consistent for high-income countries in a systematic review suggesting that GLP-1 RAs were not cost-effective compared with DPP-4 inhibitors, sulfonylureas or thiazolidinediones [66].

These economic evaluations underscore the need for country-specific strategies to enhance the cost-effectiveness of GLP-1 RAs. Such strategies entail collaborative efforts between governments and pharmaceutical companies to negotiate lower drug pricing, establish product-listing agreements and promote generic medications. Through drug price negotiations, governments and pharmaceutical companies can work together to determine equitable pricing structures that facilitate patient access to treatments without imposing excessive financial strain on the healthcare system. It is worth noting that the manufacturer of liraglutide and semaglutide has recently witnessed a significant increase in market capitalisation, reflecting the company's robust financial performance [67]. This suggests that there are realistic opportunities to negotiate lower prices for these medications without adversely impacting the company's profitability or hindering its capacity to invest in research and development.

In addition to direct price negotiations, other strategies that can be employed to achieve cost-effectiveness of GLP-1 RAs include product-listing agreements such as managed entry and risk-sharing agreements [68]. For example, the use of rebates can be implemented to adjust a drug's price according to its real-world effectiveness post approval, thus aligning the cost with the actual value delivered to patients. Another approach is tendering, which involves competitive bidding processes among pharmaceutical companies, fostering a competitive market environment that can lead to more favourable pricing outcomes for the healthcare system [69]. Once the patents for GLP-1 receptor agonists expire, it is crucial to promote and facilitate the timely development of generic alternatives or biosimilars [65]. These more affordable options can offer similar therapeutic benefits at significantly lower prices compared with the original branded drugs, alleviating the financial burden on healthcare systems. Of note, the patent for liraglutide has reportedly already expired in China and Japan, whereas patents in Europe and the USA are set to expire in 2023 [70]. If such strategies prove unsuccessful in achieving affordable drug prices or regulation agreements, governments should probably implement a hierarchical reimbursement model to prioritise reimbursement and availability of more cost-effective drugs over GLP-1 RAs, such as metformin as first-line therapy and SGLT-2 inhibitors for patients with elevated cardiovascular risk.

### Improving health literacy

Strategies to improve health literacy in people with type 2 diabetes can not only increase their understanding of the importance of preventing diabetes-related vascular complications but can also motivate them to access healthcare providers more frequently and to actively participate in the discussion with their clinician regarding treatment with potentially cardioprotective drugs including GLP-1 RAs [30, 31, 71]. By making well-informed therapeutic decisions, ideally through a shared decision-making process and the use of decision aids [30, 72], patients, particularly those with low socioeconomic status, will be more likely to individually perceive the value associated with a reduced risk for cardiovascular events and accept, to some extent, the co-payment costs of drugs with cardiovascular benefits [73]. Engaging in a meaningful interaction with their physician can also help patients address and overcome other barriers to treatment, such as concerns about subcutaneous administration, and gain the confidence needed to manage potential challenges. In the long term, this collaborative approach can enhance treatment adherence (consistently taking medication as prescribed) and persistence (continuing to refill prescriptions as required), ultimately leading to more effective treatment outcomes [73].

### Addressing physician–patient barriers

Overcoming barriers between physician and patient and the influence of structural racism is a complex issue that necessitates a multifaceted approach [30, 74]. This includes providing implicit bias training, promoting diversity within healthcare professions, and advocating for systemic changes that aim to eliminate racism and ageism within the broader scope of healthcare provision [74]. Implicit bias training can help healthcare professionals become aware of any unconscious prejudices that could potentially impact their decision making and contribute to disparities in treatment recommendations, such as the use of GLP-1 RAs. By promoting diversity within the healthcare workforce, a more inclusive environment can be created, enabling better understanding and addressing the specific needs of racial and ethnic minority individuals [74].

## Conclusion

In this review we have highlighted the disparities in the uptake of incretin-based therapies, particularly GLP-1 RAs, among individuals with type 2 diabetes from various socioeconomic backgrounds. These disparities can negatively impact the potential societal benefits of these medications in preventing diabetes-related complications. To address these inequalities effectively, an essential first step is advocating for a reduction in the price of GLP-1 RAs. This step is a prerequisite to enhance the affordability of these medications, particularly for socioeconomically disadvantaged people. Moreover, reducing the cost of GLP-1 RAs would also improve their value-for-money from a societal perspective. By prioritising cost-effective strategies, healthcare systems can foster a broader uptake and expanded use of beneficial incretin-based therapies. This approach should ideally complement other measures, which include maximising treatment benefits while minimising harms, increasing accessibility, enhancing health literacy and overcoming physician–patient barriers. It is important to note that most evidence assessing the impact of socioeconomic status on the uptake of incretin-based therapy comes from the USA, which may limit the generalisability of findings to other settings. While many socioeconomic factors and mechanisms discussed are universal, their extent and impact can vary between countries. Consequently, additional research on uptake disparities and development of context-specific strategies is crucial. Collaborative efforts to implement these strategies can boost societal benefits of incretin-based therapies and improve global outcomes for individuals with type 2 diabetes.

## Abbreviations

CVOT: Cardiovascular outcomes trial
DPP-4: Dipeptidyl peptidase 4
GLP-1 RA: Glucagon-like peptide-1 receptor agonist
SGLT-2: Sodium–glucose cotransporter 2
